# Beneficial defects: exploiting the intrinsic polishing-induced wafer roughness for the catalyst-free growth of Ge in-plane nanowires

**DOI:** 10.1186/1556-276X-9-358

**Published:** 2014-07-16

**Authors:** Luca Persichetti, Anna Sgarlata, Stefano Mori, Marco Notarianni, Valeria Cherubini, Massimo Fanfoni, Nunzio Motta, Adalberto Balzarotti

**Affiliations:** 1 Department of Materials, ETH Zurich, Hönggerbergring 64, Zürich 8093, Switzerland; 2 Dipartimento di Fisica, Università di Roma ‘Tor Vergata’, Via della Ricerca Scientifica 1, Rome 0133, Italy; 3 Dipartimento di Ingegneria dell’Impresa, ‘Mario Lucertini’, via del Politecnico 1, Rome 00133, Italy; 4 Institute for Future Environments and School of Chemistry, Physics, and Mechanical Engineering, Queensland University of Technology, Brisbane QLD 4001, Australia

**Keywords:** Nanowires, Epitaxy, Silicon, Germanium, Quantum dots

## Abstract

We outline a metal-free fabrication route of in-plane Ge nanowires on Ge(001) substrates. By positively exploiting the polishing-induced defects of standard-quality commercial Ge(001) wafers, micrometer-length wires are grown by physical vapor deposition in ultra-high-vacuum environment. The shape of the wires can be tailored by the epitaxial strain induced by subsequent Si deposition, determining a progressive transformation of the wires in SiGe faceted quantum dots. This shape transition is described by finite element simulations of continuous elasticity and gives hints on the equilibrium shape of nanocrystals in the presence of tensile epitaxial strain.

**PACS:**

81.07.Gf; 68.35.bg; 68.35.bj; 62.23.Eg

## Background

In the last few years, germanium (Ge)-based nanoelectronics is living a second youth. This renewed interest stems from recent advances in high-κ dielectrics technology compatible with Ge and has been prompted by the advantageous electrical properties of Ge compared to Silicon (Si) [1,2]. On the roadmap of continuous scaling of transistors with higher operation speed, Ge is ranked among the most promising alternate materials for integration into the Si platform, due to the high mobility and saturation velocity leading to effective device performance combined with reduced power consumption [3]. Ultrascaled Ge-based electronics nonetheless is still in its infancy, and extensive fundamental research on Ge nanofabrication is required so that these appealing semiconductor properties could compensate for the high material costs.

Novel quantum-related properties due to scaled dimensionality have stimulated the quest for fabricating one-dimensional nanostructures like nanowires (NWs) which have demonstrated great potential for applications in a variety of fields such as high-temperature thermoelectrics [4], super-efficient lithium ion batteries [5], and new-generation photovoltaics [6]. In this context, Ge NWs are particularly promising, owing to the smaller bandgap and the larger exciton Bohr radius of Ge, which provide quantum confinement effects at larger nanowire sizes compared to Si [7].

One major hurdle for technological application of NWs is to develop a growth method combining synthesis and assembly in a single step, hopefully also being compatible with traditional planar device architecture. Ge NWs are usually grown by vapor-liquid-solid (VLS) mechanism [8-10]. In this process, the metal seed, which is required as catalyst, is left in the final wire structure, and this can degrade the performance of nanowire-based devices.

In this paper, we outline a metal-free fabrication route for in-plane Ge NWs on Ge(001) substrates. We will show that, by exploiting the intrinsic polishing-induced defects of standard Ge wafers, micrometer-length wires can be grown by physical vapor deposition (PVD) in an ultra-high-vacuum (UHV) environment.

We will also show that, under epitaxial strain induced by subsequent Si deposition, the shape of the wires can be tailored, resulting in a progressive transformation of the wires in SiGe faceted quantum dots. This shape transition, which has been described by finite element (FE) simulations of continuous elasticity, gives hints on the equilibrium shape of nanocrystals in the presence of tensile epitaxial strain.

## Methods

All experiments are carried out by using commercial epi-ready, prime-grade polished Ge(001) wafers (Sb-doped with resistivity of 7 to 9 Ω cm). The samples were outgassed in UHV (*p* < 5 × 10^-11^ mbar) for several hours at 300°C. For NW synthesis, Ge(001) substrates are prepared by a mild sputtering/annealing procedure: Surface cleaning is performed by 4 cycles of Ar sputtering (830 V, 20 min) and annealing at 830°C by direct current heating. Sputtering is performed at normal incidence by a differentially pumped ion gun at a base pressure of 2 × 10^-7^ mbar. Ge and Si are deposited at 500°C by PVD using e-beam evaporators in UHV. The growth is monitored in situ by scanning tunneling microscopy (STM; Omicron VT, Omicron NanoTechnology GmbH, Taunusstein, Germany). Ex situ morphological characterization is performed by atomic force microscopy (AFM) in tapping mode (Asylum Research Cypher, Santa Barbara, CA, USA), optical (Leica DM2700M, Leica Microsystems, Wetzlar, Germany), field emission scanning electron microscopy (FE-SEM; Zeiss-SIGMA, Carl Zeiss, Inc., Oberkochen, Germany), and transmission electron microscopy (TEM; JEOL 2100 at 200 kV, JEOL Ltd., Akishima-shi, Japan). The samples for TEM characterization are prepared by ‘lift out’ technique using a focus ion beam (FIB) with Ga ions (FEI Quanta 3D, FEI, Hillsboro, OR, USA). A layer of FIB-deposited platinum is placed over the area of interest to prevent milling from damaging the surface of the TEM specimen cross-section. Two trenches are milled on either side of the tungsten that has been deposited above the area of interest in order to obtain a final thickness of the membrane between 50 and 100 nm. The membrane is then transferred to the TEM grid with a micromanipulator. Composition of strained SiGe NWs is probed by Raman spectroscopy and imaging (WITec Alpha300R, WITec Wissenschaftliche, Ulm, Germany) using 532-nm-laser excitation.

## Results and discussion

### Characterization of substrate defects after the sputtering procedure

**Figure 1:**
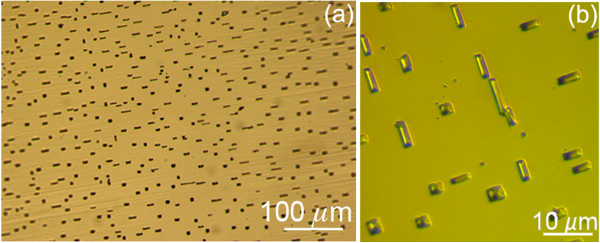
**Optical microscopy.** Optical microscopy images **(a, b)** of the Ge(001) surface after 4 sputtering/annealing cycles.

Although the majority of atomic-scale STM studies on the Ge(001) face have been performed on surfaces prepared by the ion-sputtering-based process [11], investigations of the mesoscale surface structure after sputtering are, instead, rather scattered. Nonetheless, the very peculiar orientational dependence of surface energy of Ge, with the major (001) and the (111) faces being almost equally stable [12], suggests the appearance of a non-trivial surface morphology with the ion-sputtering process. Figure 1 shows large-scale optical microscopy images of the Ge(001) surface after 4 cycles of sputtering/annealing following the procedure described in the experimental section.

**Figure 2:**
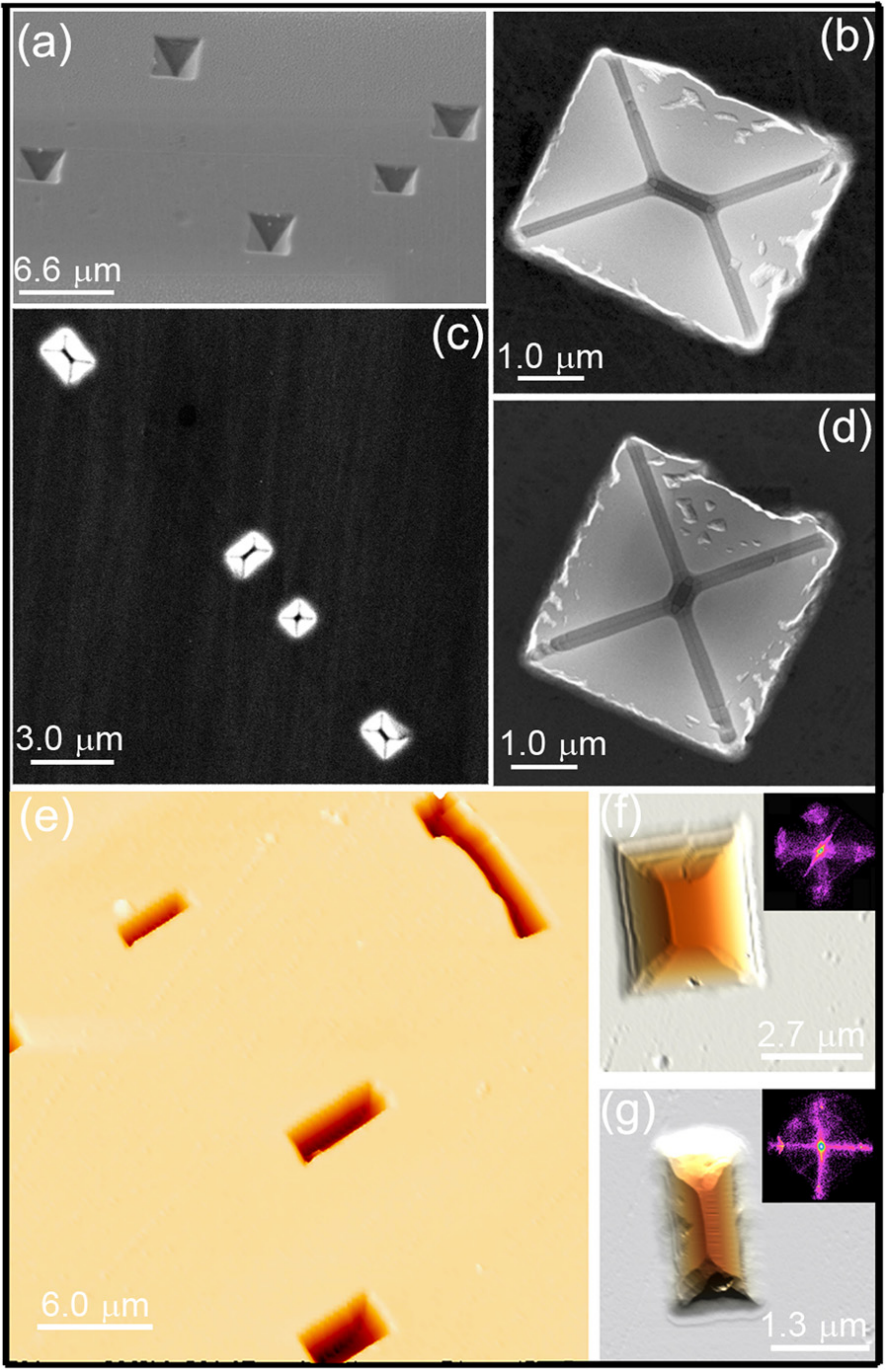
**Pit faceting. (a, b, c,d)** SEM images of the pits forming on the Ge(001) surface after 4 sputtering/annealing cycles. **(e, f, g)** AFM images showing the pit morphology. In the insets of **(f)** and **(g)**, the FPs of the corresponding images are shown.

**Figure 3:**
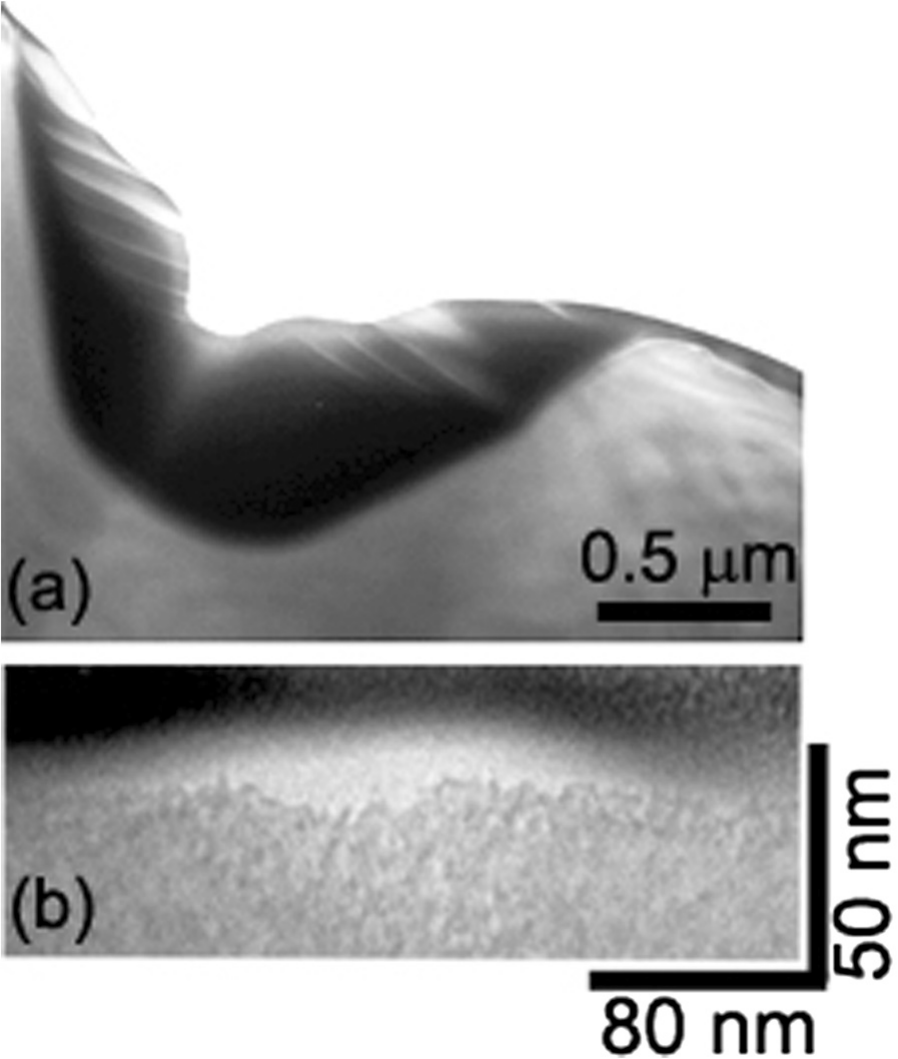
**TEM microscopy.** Cross-sectional TEM images showing: **(a)** a pit and **(b)** Ge wires grown inside a polishing-induced trench. The topmost black layer is the protective Pt film deposited for FIB cross-sectioning.

As evident, flat areas alternate with regular pits having square or rectangular shape. High-resolution SEM and AFM images displayed in Figure 2 reveal that pits are bounded by well-defined facets and indeed appear as inverted square pyramids and elongated huts. Moreover, from a statistical examination of AFM scans, it can be inferred that the lateral facets of the pits have a dominant {111} orientation. This distinct faceting can be readily visualized by applying an image-analysis tool known as facet plot (FP) to AFM images [13]. It consists of a two-dimensional histogram displaying the component of the surface gradient on the horizontal and vertical axes: Faceting thus produces well-defined spots in the FP. In the case of the histograms shown in the insets of Figure 2f,g, the four major spots correspond to a polar angle of approximately 55° from the (001) plane, i.e., to {111} faces. {111}-faceting is also confirmed by cross-sectional TEM measurements (Figure 3a).

The observed extended {111} faceting can be explained by the surface roughening induced by the sputtering process: This produces a variety of unstable surface orientations which, during the subsequent annealing, collapse into the closest stable crystal face. Since the (001) and the (111) faces have roughly the same surface energy in Ge [12], the faceting does not lead to a unique dominant surface orientation, but, conversely, results in the coexistence of the two crystal facets. Indeed, the formation of similar inverted pyramids has been observed during the growth of thick Ge(001) films [14,15].

**Figure 4:**
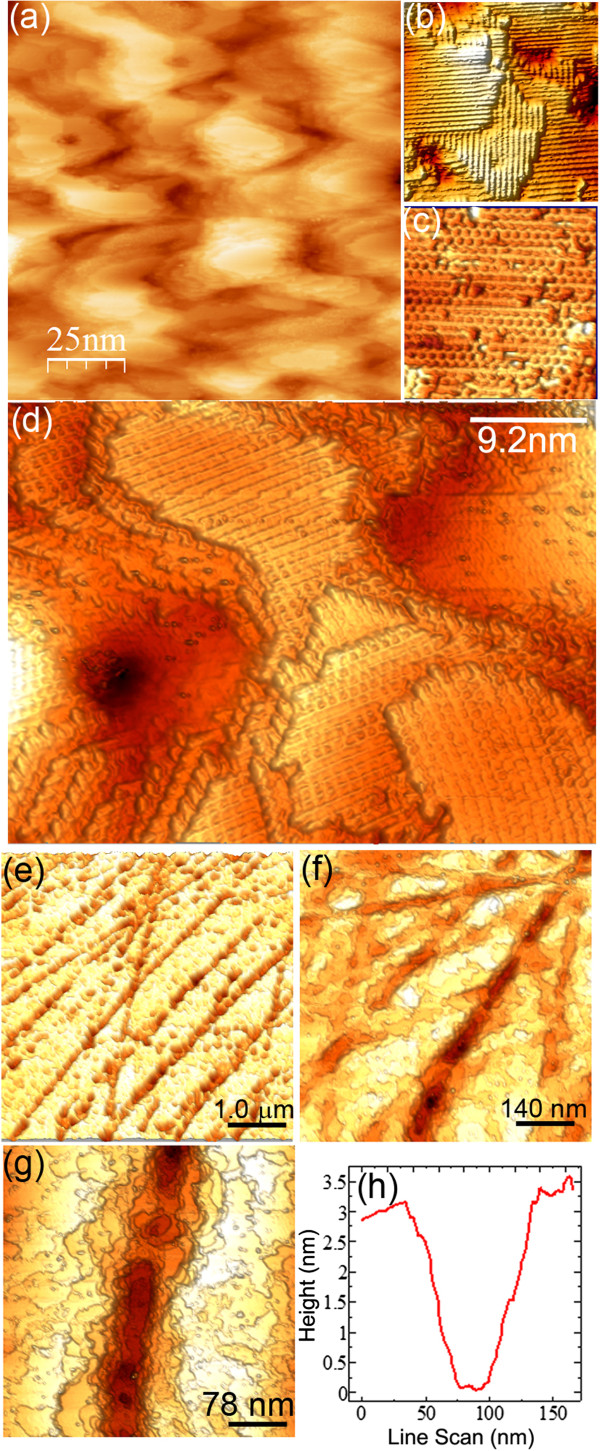
**STM imaging.** STM images of **(a, b, c, d)** the reconstructed Ge(001) surface and **(e, f, g)** the polishing-induced trenches. The size of panels **(b)** and **(c)** is, respectively, 31 × 31 nm^2^ and 18 × 18 nm^2^. In **(h)**, the line profile of the trench reported in **(g)** is shown.

Notably, this scenario is almost impossible to grasp within the length scale probed by STM: Down to the atomic scale, the surface shows the usual atomic ordering consisting in flat reconstructed terraces with *c*(4 × 2)/(2 × 1) domain patterns and atomic steps (Figure 4a,b,c,d) [11], whereas the resulting pit areas are too steep for STM imaging.

Interestingly, between the atomic length scale and micrometer-size features like the pits, we discovered other characteristic defects of the substrate surface. Their presence is hinted in Figure 1a as shallow dark stripes running across the whole imaged area. The detailed morphology of these features is shown by STM measurements (Figure 4e,f,g,h): They appear as shallow trenches with a depth of a few nanometers and an average width of about 100 nm, as shown by the cross-sectional profile in Figure 4h. Their length is instead much longer and can also reach several hundreds of microns. We found that these trenches are already present on the bare substrate before sputtering. Comparison with very similar images observed in literature on diverse substrates [16-18] sheds light on the origin of these almost one-dimensional features. These are the results of the residual polishing-related damage of Ge wafers which are usually observed at this length scale, despite the mirror-like surface after mechanical polishing. We found that 4 cycles of sputtering/annealing cleaning only partially smooth away this mesh of trenches, reducing their height by about 50% and resulting in the shallow imprints displayed in Figure 4. After 8 cycles, this polishing-related roughness is instead entirely washed out. Similarly, the trenches are smoothed down completely by a wet chemical etching processes, i.e., oxide stripping in HCl/H_2_O followed by passivation in H_2_O_2_/H_2_O [19,20]. A comparison of the large-scale morphology obtained by different surface treatments is shown in Additional file 1.

### Exploiting polishing-induced defects for the growth of Ge nanowires

**Figure 5:**
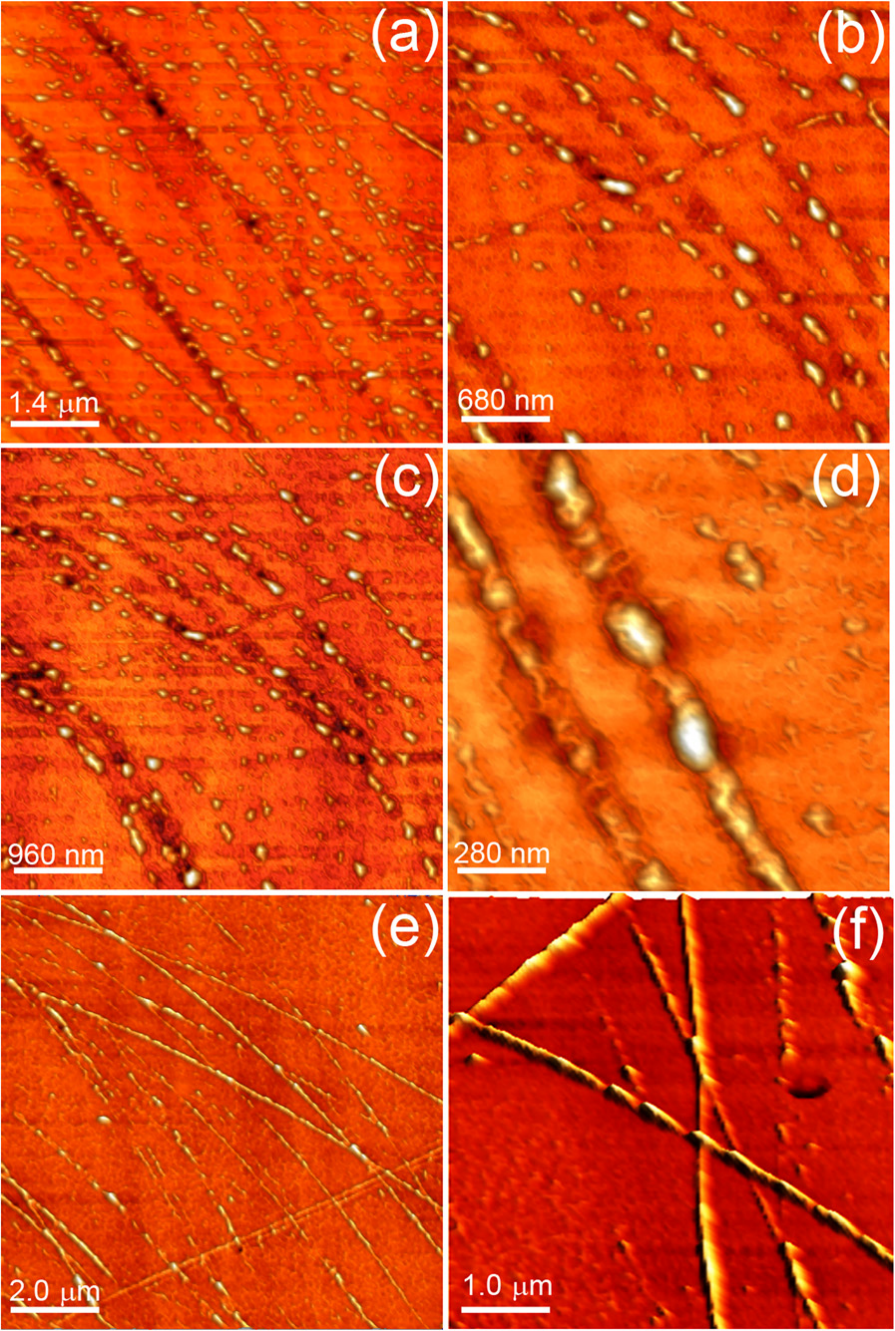
**Wire formation. (a, b, c, d, e, f)** STM images showing different stages of the formation of the wires. The total Ge coverage is 12 MLs.

**Figure 6:**
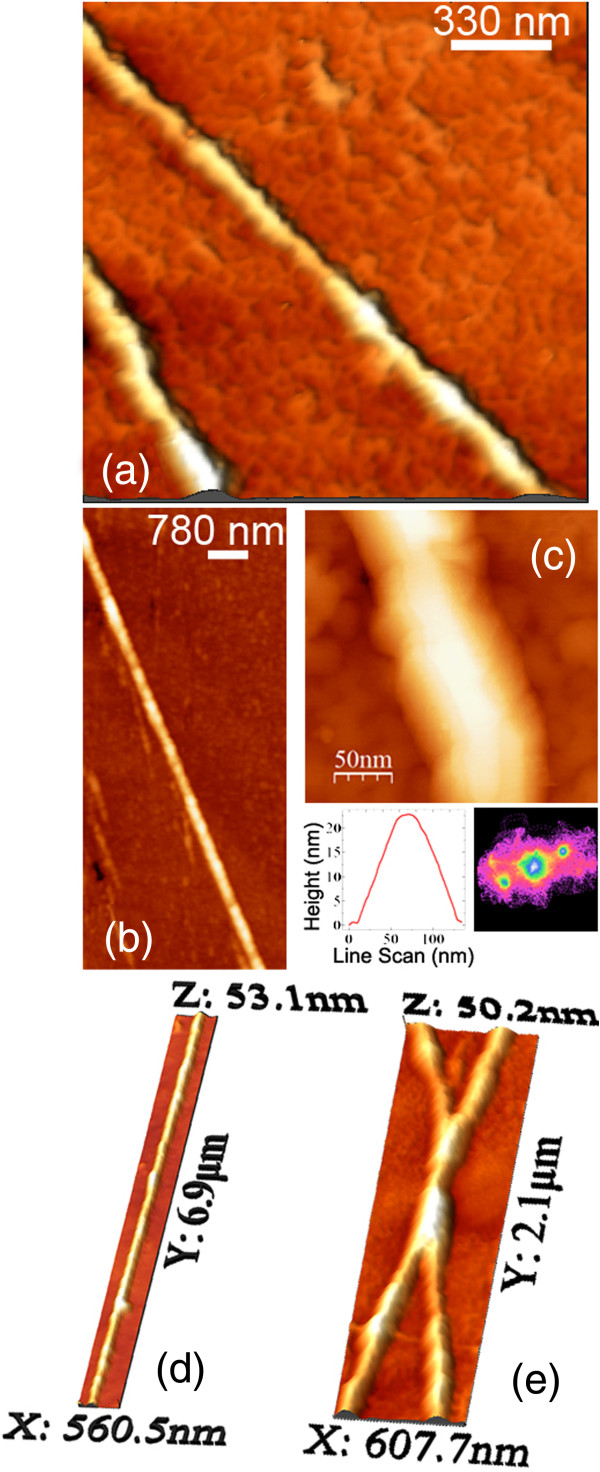
**Wire faceting. (a, b, c, d, e)** STM images showing the morphology of the wires. The bottom insets of **(c)** show, respectively, (left panel) the line profile and (right panel) the FP of the wire in **(c)**.

It is known that the homoepitaxial growth of Ge on Ge(001) can hardly be reduced to the classical picture of layer-by-layer growth mode: A complex interplay between thermodynamic stability and kinetic diffusion bias [21-23] leads to the formation of three-dimensional structures such as mounds and islands. We now show how the shallow trenches can be positively exploited for guiding the self-assembly of three-dimensional structure and orienting the formation of in-plane Ge nanowires during the growth of a Ge overlayer. In Figure 5, different stages of the growth have been imaged by in situ STM, up to a final Ge coverage of 12 monolayers (MLs). It can clearly be seen that three-dimensional structures selectively form inside the trenches; the three-dimensional mounds grow and coalesce until the whole trench is completely filled up, leading to the formation of a long in-plane wire. High-resolution images, displayed in Figure 6, reveal that the wires are bounded by lateral {113} facets.Moreover, following the underlying mesh of the trenches, the wires show micrometer-length straight sections (Figure 6d) which alternate with junction nodes (Figure 6e). Cross-sectional TEM measurements clearly confirm the presence of the shallow trenches under the wires (Figure 3b) and also show the absence of any subsurface dislocation defect close to the substrate/wire interface. This indicates that only the presence of the trench is enough to bias the growth of Ge to heterogeneous nucleation.

**Figure 7:**
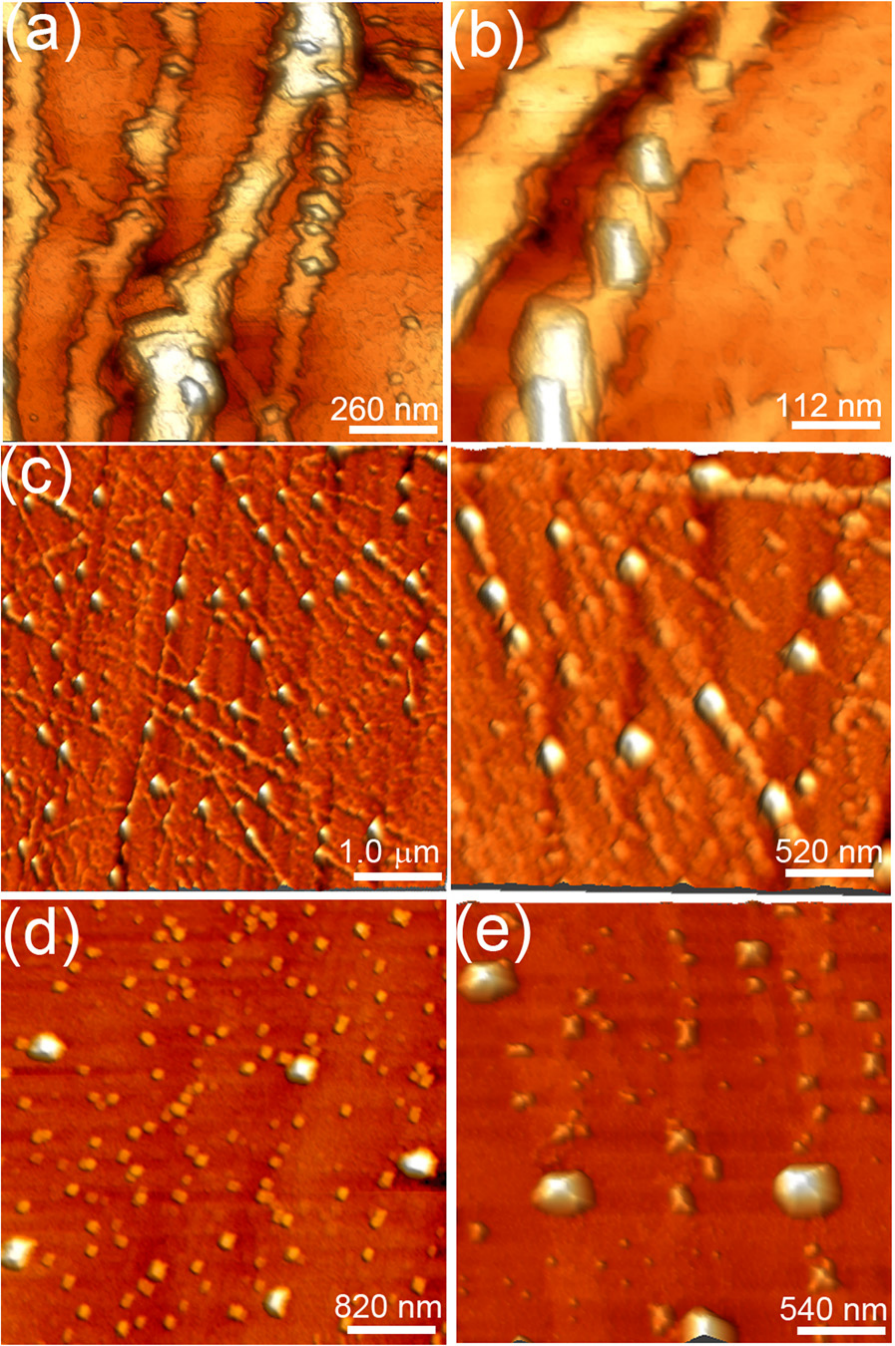
**Wire to dot transition. (a, b, c, d, e)** STM images showing different stages of the wire-to-dot shape transition induced by Si deposition. The total Si content, obtained by Raman spectroscopy, is 10%.

**Figure 8:**
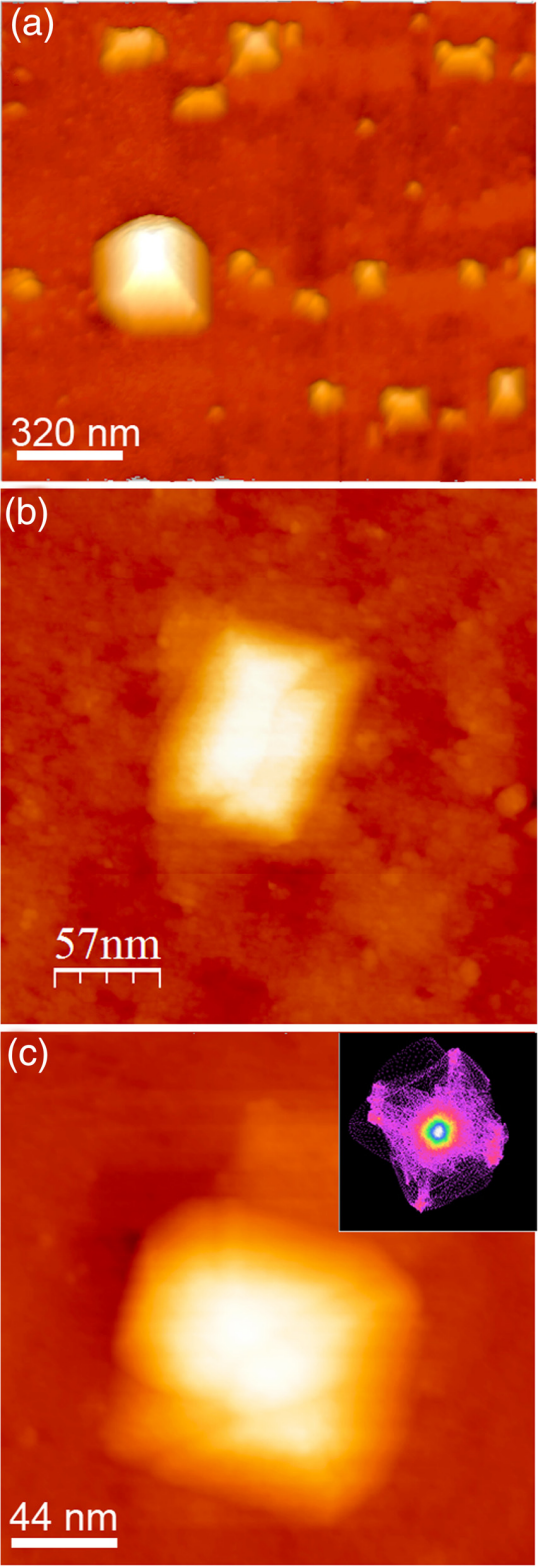
**Dot faceting. (a, b,c)** STM images showing the morphology of the SiGe dots. In the inset of **(c)**, the FP of the corresponding image is reported.

**Figure 9:**
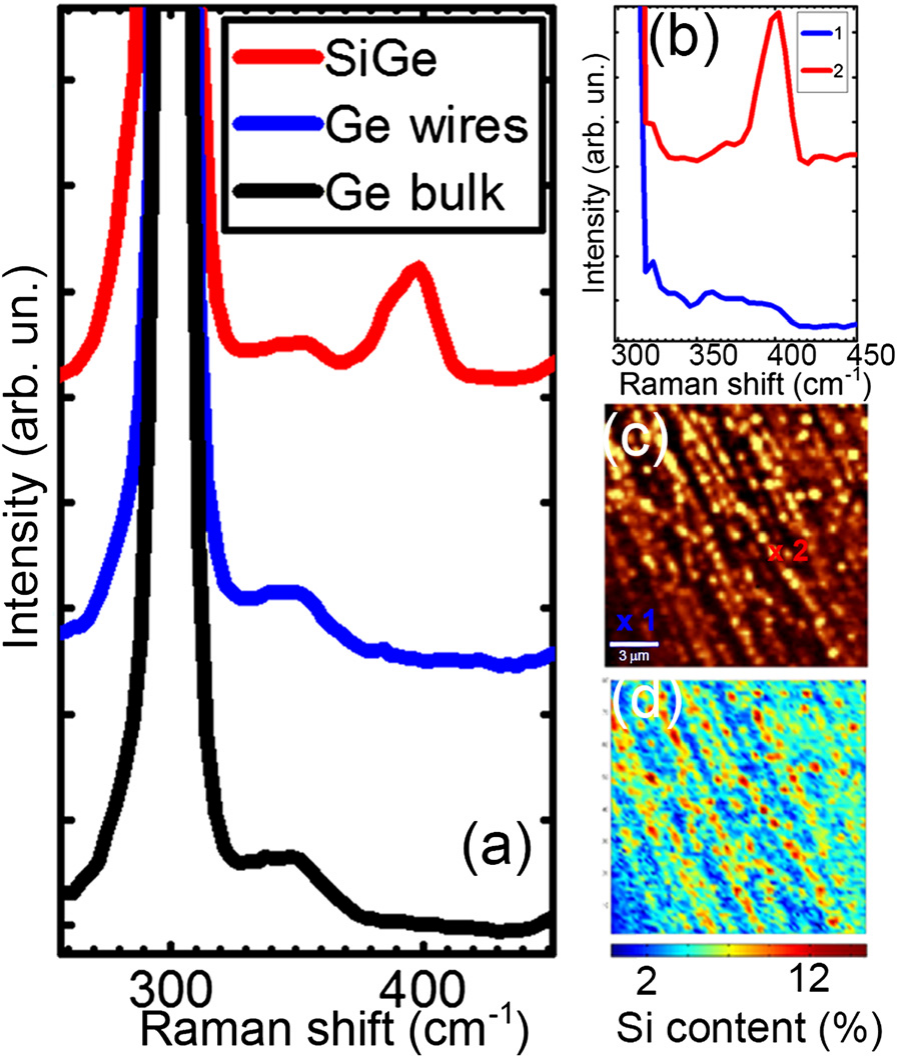
**Raman spectroscopy. (a)** Raman spectra of bare Ge(001) substrate, Ge wires, and SiGe islands formed from the wires with Si deposition. **(b)** Spectra extracted from the Raman image shown in **(c)**. **(c)** Raman image. The color scale gives the intensity of the SiGe alloy peak at 399 cm^-1^. The markers highlight the position of the spectra reported in **(b)**. **(d)** Composition image obtained from **(c)** by applying the relative-intensity method described in the text.

Being the result of homoepitaxial growth, the wires are totally strain-free. We now show that epitaxial strain introduced by Si deposition dramatically alters the growth morphology, determining a shape transition from wires to dots. As soon as Si is deposited, we notice the formation of faceted squared and rectangular dots along the wires (Figure 7). These dots progressively grow at the expense of the wires, until the latter completely disappear. By carefully analyzing the STM images of the dot assembly, it is still possible, however, to notice the residual imprint of the wires, appearing as a shallow mound along which the dots are aligned (Figure 7e). Table 1 summarizes the morphological parameters of wires and dots obtained from a statistical analysis of STM and AFM images. It can be noticed that, during the shape transition, the total volume of nanostructures is preserved: The micrometer-long wires are replaced by a large number of dots, which show a bimodal size distribution. By inspecting in details the morphology of the dots (Figure 8), it can be seen that the islands are either squared or elongated pyramids (huts), again bounded by {113} facets, as indicated by the FP analysis (Figure 8c).This suggests that the observed shape change is not driven by the appearance of new stable facets with strain, but rather by a more efficient strain relaxation or a better surface/elastic energy gain which favors the islands over the wires. Before discussing the island/wire stability, it is interesting to estimate the Si content which is needed to activate the shape transition. Figure 9a shows Raman spectra measured, respectively, on a bare Ge(001) substrate, on a wire-covered substrate, and on an island-covered substrate after the shape change activated by Si deposition.

**Table 1:** Morphological parameters of wires and dots

	**Total volume [measured on a 4 × 4** **μm**^**2**^**image] (nm**^**3**^**)**	**Average height (nm)**	**Average lateral size**^**a**^**(nm)**	**Surface (*S*) to volume (*V*) ratio** ***S*/*V***^**2/3**^
Wires	(2.0 ± 0.5) × 10^7^	18 ± 5	100 ± 10	10.3
Dots	(1.8 ± 0.5) × 10^7^	40 ± 5^b^	230 ± 10^b^	5.5
		15 ± 5	130 ± 10	

^a^The width of the wires and the island edge size is reported. ^b^Dots show a bimodal distribution.

As expected, the bare and the wire-covered substrate show almost identical spectra in which the only feature is the Ge-Ge band located at about 300 cm^-1^. Conversely, the island-covered sample shows an extra peak at about 399 cm^-1^, being the Si-Ge alloy band. The band associated to the Si-Si mode cannot be detected, also within an extended energy range, as expected for low Si contents [24]. In fact, the Si content *x*, estimated by the relative intensities of the Ge-Ge and the Si-Ge bands [25], i.e., *I*_Ge–Ge_/*I*_Si–Ge_ *=* 1.6(1 - *x*)*x*^-1^, is *x* = 0.1. Therefore, a very small quantity of Si is indeed enough to drive the wire to island shape change. This can be only explained if the deposited Si does not cover the surface uniformly, but rather concentrates into the wires. In order to validate this hypothesis, we exploited Raman imaging. A complete spectrum is acquired at each and every pixel of the image, and then, a false color image is generated based on the intensity of the Si-Ge mode. Figure 9b shows two spectra extracted from the marked position on the Raman image displayed in panel c. In Figure 9d, we report the corresponding composition image obtained by the relative intensity method. As shown, the Si is totally absent from the substrate among the wires, whereas in the wires, it is intermixed with Ge. Besides, it can be seen how the brighter pixels, corresponding to Si-rich areas, exactly define the wire shape. Moreover, we also see many bright spots which are the dots forming along the wires.

**Figure 10:**
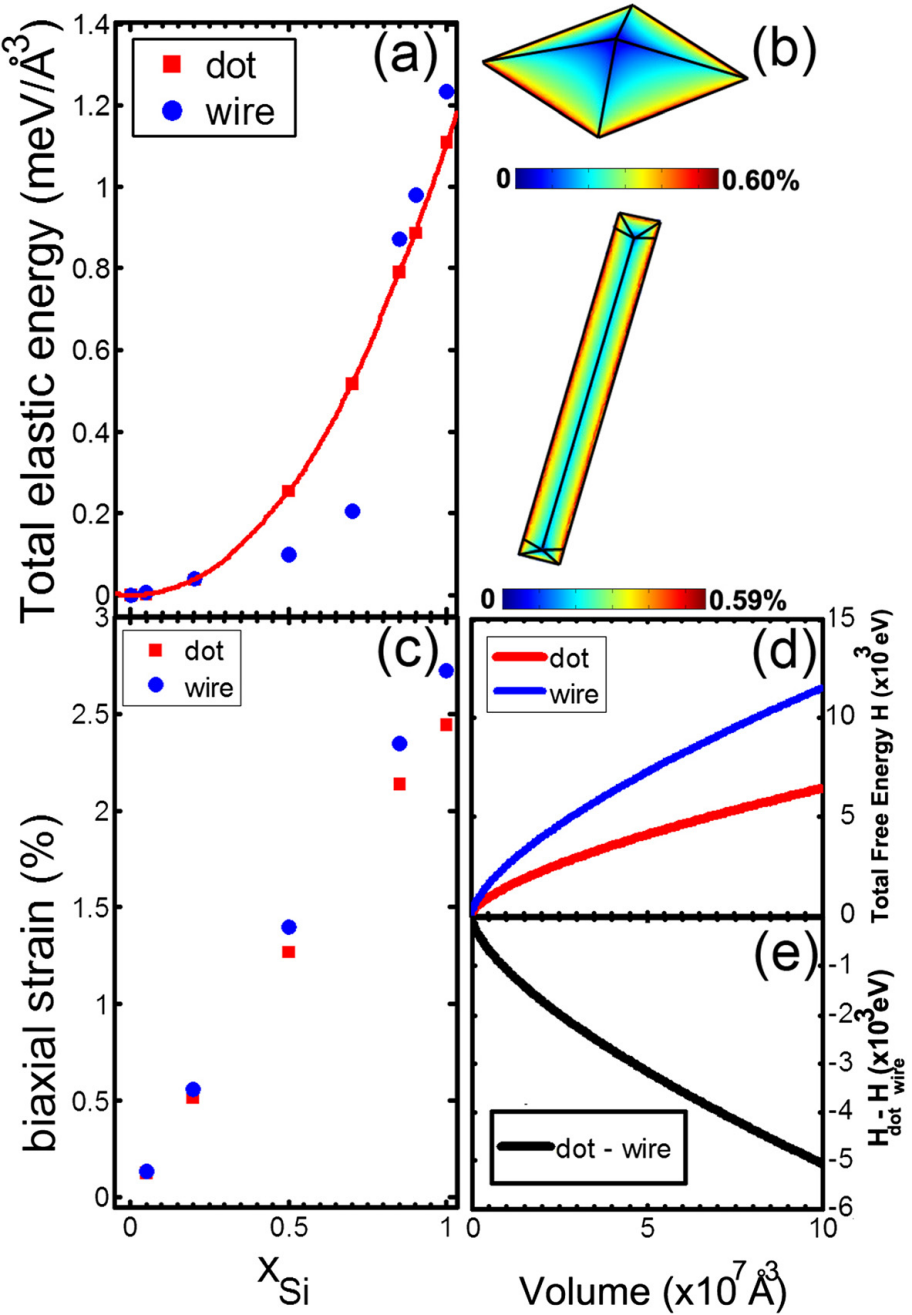
**FE simulations. (a)** Total elastic energy of wires and dots as a function of the Si content. **(b)** Three-dimensional maps of biaxial strain for pyramidal dots and wires for a Si content of 10%. **(c)** Average biaxial strain for wires and dots as a function of the Si content. **(d)** Total strain + surface energy for wires and dots as a function of volume. **(e)** Relative difference of the curves shown in **(d)**.

In order to better understand the wire-to-island transition, we modeled the elastic properties of the system by FE simulations within continuum elasticity theory (Figure 10) [26].

Wires and islands were modeled by realistic three-dimensional geometries (sketched in Figure 10b), for a Si composition ranging between 0 and 1. Both wires and islands have been assumed to be bounded by {113} facets and grown on a Ge(001) substrate. The aspect ratios of dots/wires were taken from STM measurements. Figure 10a shows the composition dependence of the total elastic energy density *e*_relax_ for wires and islands. *e*_relax_ is the residual strain energy stored in a SiGe island(wire) and in the Ge substrate after relaxation and normalized to the island(wire) volume. As evident, the dots and the wires show almost the same elastic energy density for low Si contents, whereas the elastic energy of the dots becomes lower for *x* ≳0.75. Indeed, Figure 10c shows that, at high Si concentration, the strain relaxation is more efficient for the dots. The residual tensile strain obtained from FE calculations for a Si content *x* = 0.1, i.e., the composition determined by Raman spectroscopy, is found to be *ε* = +0.27%. To validate the model, it is interesting to compare this value with an experimental estimate of the strain. It is well-known the frequency position of the Si-Ge Raman mode depends on the residual biaxial strain as [27]

(1) $$\omega_{\mathrm{Si}‐\mathrm{Ge}}\left(\varepsilon \right)=400.1-570\varepsilon$$

By using the position of the SiGe alloy peak determined in our spectra, i.e., *ω*_Si *-*__Ge_ = 398.6 cm^-1^, we obtained a residual strain of +0.25%, a value which closely matches the result of the simulations.

In order to discuss the relative stability of dots and wires, the strain energy term has to be combined with the surface energy contribution to define the total-energy gain associated to the formation of a three-dimensional dot/wire of volume *V*, namely

(2) $$E_{\mathrm{tot}}=\left(e_{\mathrm{relax}}-e_{\mathrm{WL}}\right)V+\left(\gamma_{S}C_{S}-\gamma_{B}C_{B}\right)V^{2/3}$$

where *e*_WL_ is the strain energy density of a flat pseudomorphic Si_0.1_Ge_0.9_ film grown on Ge(001), *γ*_*S*_ and *γ*_*B*_ are, respectively, the surface energies of the lateral {113} facets and of the Ge(001) face of the substrate. *C*_*S*_ = *SV*^-2/3^ and *C*_*B*_ = *BV*^-2/3^ are shape-dependent factors which depend on the relative extension of the area of the lateral facets, *S*, and of the base area, *B*, of dots/wires. Previous results have shown that both the tensile strained Ge(113) [28] and the Ge(001) [29] surfaces have roughly the same surface energy value of about 65 meV/Å^2^; therefore, for the sake of simplicity, we assume *γ*_*S*_ = *γ*_*B*_ = 65 meV/Å^2^. Figure 10d shows the dependence of the total energy of dots/wires on the volume; in panel (e), their relative difference is plotted. As evident, within the experimentally significant volume range, dots are always more stable than wires. This is due to their lower surface area per unit volume (about 40% less) compared to the wires (Table 1). The measured surface to volume ratios match well with those expected for ideal {113} wires and islands. The analysis, thus, confirms that the wires are metastable structures which are formed solely due to the presence of the preexisting polishing-induced defects. In the presence of tensile epitaxial strain induced by Si deposition, the wires thus evolve into the stable dot shape which allows a more efficient strain relaxation.

## Conclusions

In summary, we have described the quite complex mesoscale structure of Ge(001) substrates cleaned by sputtering/annealing treatments, indentifying the sputtering-induced defects and distinguishing them from polishing-induced intrinsic defects. By positively exploiting the polishing-induced defects of standard-quality commercial Ge(001) wafers, micrometer-length Ge wires can be grown without introducing any metal catalyst. The shape of the wires can be tailored by the epitaxial strain induced by subsequent Si deposition, determining a progressive transformation of the wires in SiGe faceted quantum dots. We remark that the spatial distribution of the wires (i.e., direction, spatial ordering, etc.), and therefore of the dots formed by Si overgrowth, are dictated by the characteristics of the polishing-induced trenches. As a future perspective, controlling the polishing feature will therefore enhance the spatial ordering of nanostructures.

## Competing interests

The authors declare that they have no competing interests.

## Authors’ contributions

LP conceived of the study and carried out its design, realization, and coordination during all the different stages; he also drafted the manuscript. AS and SM participated in the sample growth and morphological characterization. MN carried out the SEM, TEM, and Raman measurements. VC participated in the sample growth and characterization. MF, NM, and AB participated in the design and coordination of the study and helped to draft the manuscript. All authors read and approved the final manuscript.

## Supplementary Material

Additional file 1 **Surface morphology obtained by different cleaning treatments.** Comparison of large-scale surface morphology obtained by different cleaning procedures: (a) 4 cycles Ar sputtering (830 V, 20 min, 2 × 10^-7^ mbar Ar) and subsequent annealing at 830°C for 20 min. (b) 8 cycles Ar sputtering (830 V, 20 min, 2 × 10^-7^ mbar Ar) and subsequent annealing at 830°C for 20 min. (c) Ex situ chemical passivation followed by an in situ heating procedure. A GeOx passivation layer is chemically grown ex situ by a wet treatment consisting of a HCl/H_2_O 36:100 bath and subsequent H_2_O_2_/H_2_O 7:100 bath to strip/reform a GeOx passivation layer. The samples are then outgassed in situ at 230°C for 1 h, flash annealed at 760°C for 60 s to remove GeOx, and slowly cooled from 600°C to room temperature. Click here for file
