# Supplementary material for: Beneficial defects: exploiting the intrinsic polishing-induced wafer roughness for the catalyst-free growth of Ge in-plane nanowires
Source: Nanoscale Res Lett. 2014 Jul 16;9(1):358. doi: 10.1186/1556-276X-9-358 (PMC4119939; doi:10.1186/1556-276X-9-358)
Supplement: Additional file 1 — Surface morphology obtained by different cleaning treatments. Comparison of large-scale surface morphology obtained by different cleaning procedures: (a) 4 cycles Ar sputtering (830 V, 20 min, 2 × 10^-7^ mbar Ar) and subsequent annealing at 830°C for 20 min. (b) 8 cycles Ar sputtering (830 V, 20 min, 2 × 10^-7^ mbar Ar) and subsequent annealing at 830°C for 20 min. (c) Ex situ chemical passivation followed by an in situ heating procedure. A GeOx passivation layer is chemically grown ex situ by a wet treatment consisting of a HCl/H2O 36:100 bath and subsequent H2O2/H2O 7:100 bath to strip/reform a GeOx passivation layer. The samples are then outgassed in situ at 230°C for 1 h, flash annealed at 760°C for 60 s to remove GeOx, and slowly cooled from 600°C to room temperature. [file 1556-276X-9-358-S1.pdf]

Surface morphology obtained by different cleaning treatments

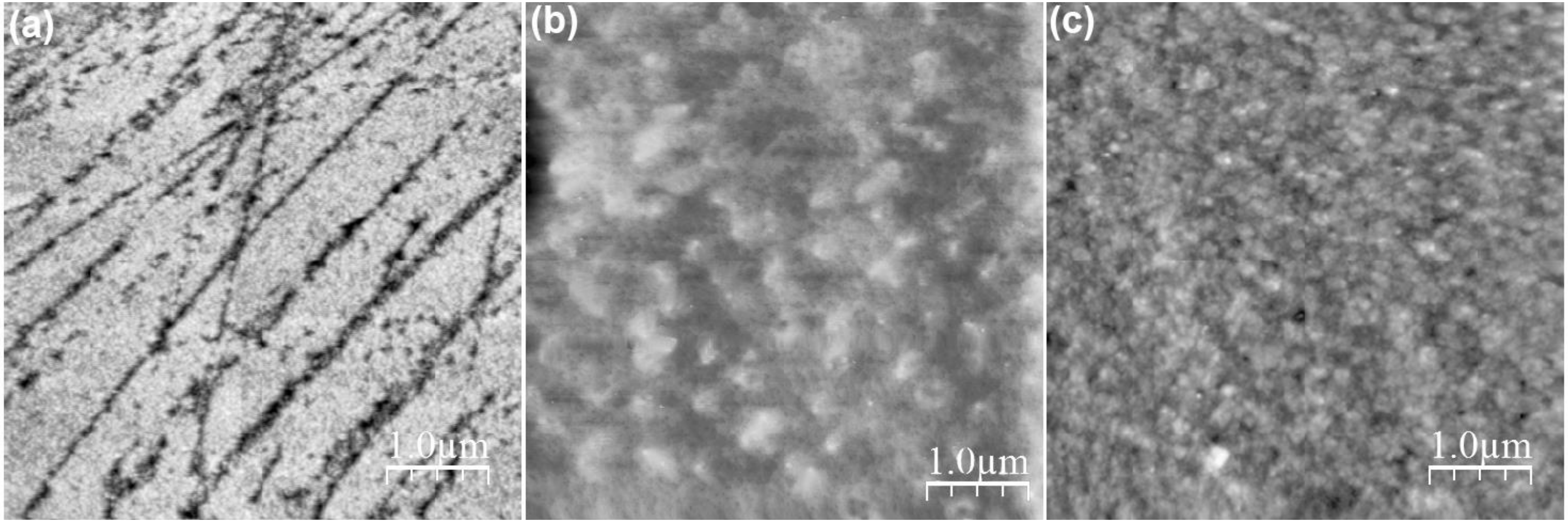

(a)
4 cycles
sputterig+annealing
830°C 20 min

(b)
8 cycles
sputterig+annealing
830°C 20 min

(c)
Ex-situ HCl:H_2O 36:100
bath and subsequent
H_2O_2:H_2O 7:100
In situ annealing 760°C
